# Identification of a Novel Five-Gene Signature as a Prognostic and Diagnostic Biomarker in Colorectal Cancers

**DOI:** 10.3390/ijms23020793

**Published:** 2022-01-12

**Authors:** Souvik Ghatak, Syrina F. Mehrabi, Lubna M. Mehdawi, Shakti Ranjan Satapathy, Anita Sjölander

**Affiliations:** Cell and Experimental Pathology, Department of Translational Medicine, Lund University, 205 02 Malmö, Sweden; souvik.ghatak@med.lu.se (S.G.); syrina.mehrabi@med.lu.se (S.F.M.); lubna.mehdawi@med.lu.se (L.M.M.); shakti_ranjan.satapathy@med.lu.se (S.R.S.)

**Keywords:** *BDNF*, *PTGS2*, *GSK3B*, *HPGD*, *CTNNB1*, prognosis, diagnosis, colon cancer

## Abstract

Colorectal cancer (CRC) is one of the leading causes of cancer-related mortality worldwide. The current TNM (Tumor, Node, and Metastasis) classification approach is suboptimal in determining the prognosis of CRC patients. The prognosis for CRC is affected by a variety of features that are present at the initial diagnosis. Herein, we performed a systematic exploration and established a novel five-panel gene signature as a prognostic and early diagnosis biomarker after performing differential gene expression analyses in five independent in silico CRCs cohort and independently validating it in one clinical cohort, using immunohistochemistry. Four genes (*BDNF*, *PTGS2*, *GSK3B*, and *CTNNB1*) were significantly upregulated and one gene (*HPGD*) was significantly downregulated in primary tumor tissues compared with adjacent normal tissues throughout all the five in silico datasets. The univariate CoxPH analysis yielded a five-gene signature that accurately predicted overall survival (OS) and recurrence-free survival (RFS) in the in silico training (AUC = 0.73 and 0.69, respectively) and one independent in silico validation cohort (AUC = 0.69 and 0.74, respectively). This five-gene signature demonstrated significant associations with poor OS in independent clinical validation cohorts of colon cancer (CC) patients (AUC = 0.82). Intriguingly, a risk stratification model comprising of the five-gene signature together with TNM stage and gender status achieved an even superior AUC of 0.89 in the clinical cohorts. On the other hand, the circulating mRNA expression of the upregulated four-gene signature achieved a robust AUC = 0.83 with high sensitivity and specificity as a diagnosis marker in plasma from CRC patients. We have identified a novel, five-gene signature as an independent predictor of OS, which in combination with TNM stage and gender offers an easy-to-translate and facile assay for the personalized risk-assessment in CRC patients.

## 1. Introduction

Colorectal cancer (CRC) is one of the leading causes of cancer-related mortality worldwide [1]. The survival of patients is closely related to the tumor stage at the time of diagnosis, as five-year survival rates range from approximately 80% for early-stage disease to 63% for late-stage disease [2]. To limit the potential side effects and unnecessary health care expenditure, the most common treatment for low-risk stage I and stage II colon cancer (CC) patients is curative surgery alone, and adjuvant therapy is only recommended to those with high-risk stage II and stage III–IV tumors [3,4]. However, the prognosis of low-risk patients who have undergone curative surgery is variable, since relapse is known to occur in a small fraction of these patients [5,6]. Therefore, identifying these relapse-prone patients could greatly contribute to the optimization of treatment selection. Furthermore, chemotherapy is only effective in a few patients, while some patients respond initially but will become resistant later. Therefore, there is an urgent need to find overall survival (OS) and recurrence-free survival (RFS) biomarkers to increase the mortality rate. Finding sensitive diagnostic and prognostic biomarkers is an urgent need for CRC patients. The prognosis for CRC is affected by a variety of features that are present at the initial diagnosis. While colonoscopy screening remains the most reliable diagnostic tool for CRC patients, it is necessary to develop a noninvasive biomarker for the early diagnosis of CRC patients before colonoscopy. The treatment of CRC fails if the tumors are aggressive at the time of diagnosis. Therefore, until now, it has been extremely important to find and improve the variables that define patient prognosis in the early stages of the disease. On the other hand, the availability of noninvasive, cost-effective, and highly accurate biomarkers is urgently needed to increase patient participation in affordable screening programs and facilitate the detection of early-stage CRCs. In contrast, recent reports have shown that noninvasive screening strategies such as cell-free circulating biomarkers are acceptable and feasible, as high-throughput molecular technology has rapidly developed in the past decade. An accurate and robust gene signature can be of great help for a more precise diagnosis of the disease in clinical practice.

Brain-derived neurotrophic factor (BDNF) is a protein belonging to neurotrophins, a family of growth factors that mediate neuronal migration, development, and differentiation via its tyrosine kinase receptors tropomyosin receptors TrkA, TrkB, and TrkC [7]. The participation of BDNF and its TrkB receptor in oncogenesis has been the subject of many studies in recent years [8,9]. BDNF has many different carcinogenic effects via its receptor TrkB, inducing epithelial–mesenchymal transition-like transformation, anoikis resistance, and metastasis [10]. It has also been shown in head and neck cancer that BDNF induces RAS activation via TrkB [11]. In our recent finding, we have shown that BDNF also has an important role in CRC in a mice xenograft model and that BDNF mRNA and protein expression can be used as a prognostic marker for CRC patients [12,13].

Building upon this evidence, we have performed an unbiased, systematic, and comprehensive genome-wide discovery to identify a novel and robust gene signature (gene selection based on the significant correlation with *BDNF* and significant association with cancer pathology) that can predict tumor recurrence in patients with stage I, II, and III CRC. By analyzing multiple clinical cohorts that included a total of 1 987 CRC patients, we demonstrate that this selected gene signature overall and recurrence classifier has superior predictive power over clinicopathologic risk determinants and currently available commercial assays, and this combined with its robust performance even in FFPE tissues makes it attractive for relatively immediate clinical translation. Furthermore, we performed a comprehensive analysis of transcriptome profiling data and identified a novel signature for the early diagnosis of CRC patients.

## 2. Results

**Figure 1:**
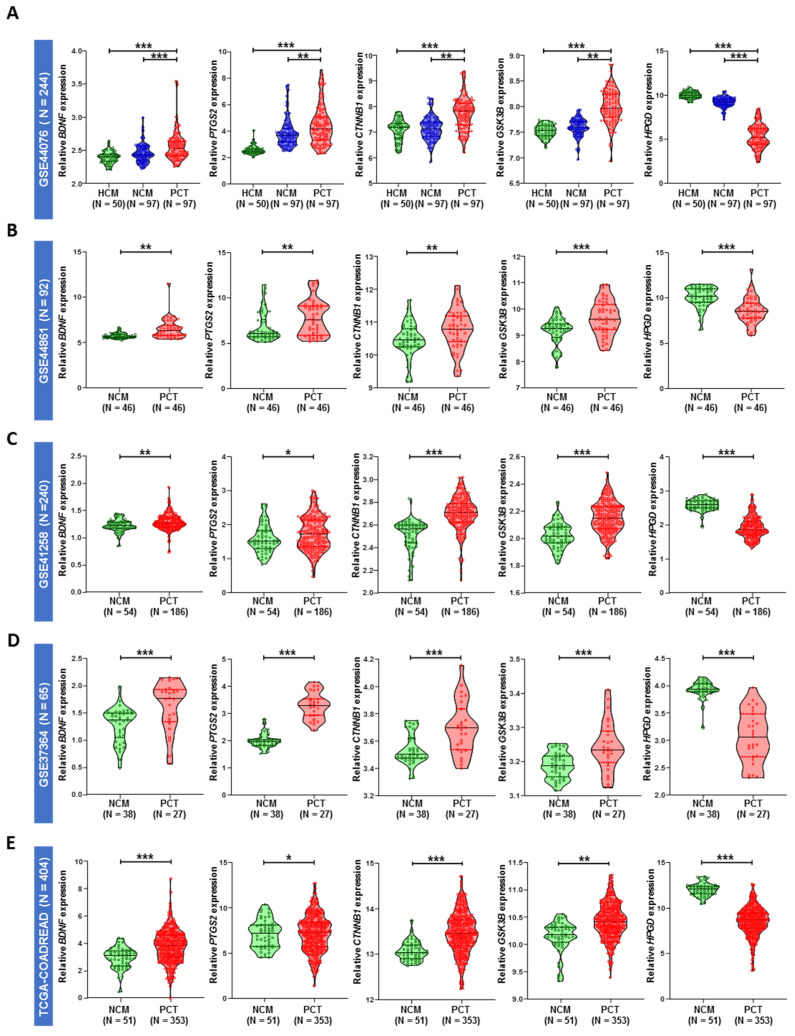
Violin plot of the relative expression of five selected genes (*BDNF*, *PTGS2*, *CTNNB1*, *GSK3B*, *HPGD*) in the microarray and RNA-seq datasets. (**A**) GSE44076, (**B**) GSE44861, (**C**) GSE41258, (**D**) GSE37364, (**E**) TCGA-COADREAD. HCM—Healthy control mucosa; NCM—Normal colon mucosa (matched); PCT—Primary colon tumor (matched). *p* value * < 0.05, ** < 0.01, *** < 0.001.

We employed a Cox proportional hazard regression model and forward selection method to identify a five-gene signature that can predict five-year OS for CC or CRC patients based on the in silico discovery and validation gene microarray and RNAseq datasets of five different cohorts (Figure 1A–E). To further evaluate the performance of this gene signature, one TMA-based clinical cohort for prognostic assessment and plasma from CRC patients for diagnostic assessment were subsequently validated.

### 2.1. Identification of Genes Associated with OS and RFS in the Training and Validation Cohorts

Our data included expression values from five different in silico microarrays and RNAseq datasets from the GEO and TCGA-COADREAD databases. Each sample had observed (RFS or censoring) time and censoring status. A total of five cancer-related genes (*BDNF*, *PTGS2*, *CTNNB1*, *GSK3B*, and *HPGD*) were selected based on the Gene Ontology database. In silico discovery cohorts were used to identify the differential expression of these five genes between adjacent normal and tumor samples in CRC patients. For prognostic assessment, a total of 32 dysregulated genes associated with OS were initially identified from the discovery cohort (*p* < 0.05), including these five genes. Among the five genes, four genes (*BDNF*, *PTGS2*, *CTNNB1*, and *GSK3B*) were uniformly and significantly upregulated, and one gene (*HPGD*) was downregulated throughout all five datasets (Figure 1). All the five genes can predict the patient’s prognosis significantly in univariate analysis in discovery TCGA-COADREAD and in silico validation GSE39582 database (Figures S1 and S2).

**Figure 2:**
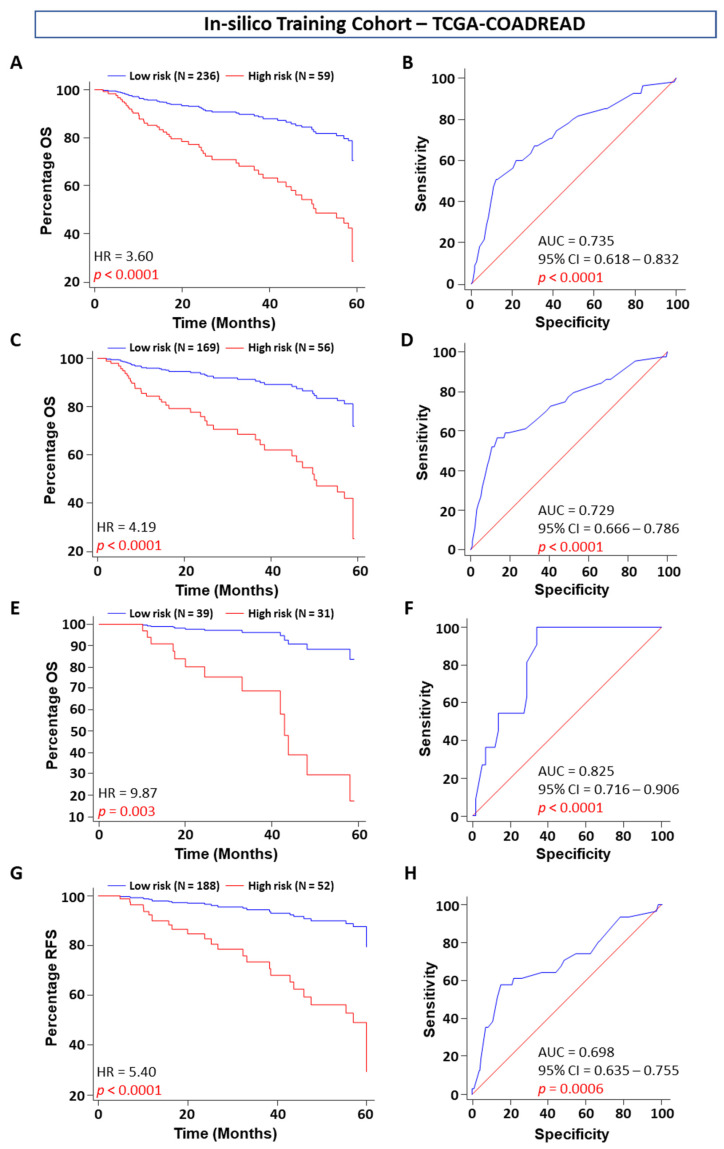
Kaplan–Meier survival plots and receiver operating curves (ROCs) for OS and RFS stratified by five-gene signature scores in the in silico training cohort (TCGA-COADREAD). (**A**) Five-gene signature OS (**A**) and ROC (**B**) curves for low- and high-risk patients. Five-gene signature OS, ROC curves for colon (**C**,**D**) and rectum (**E**,**F**) cancer patients group, respectively, and (**G**) five-gene signature RFS (**G**) and ROC (**H**) curves for low- and high-risk patients.

Using the five-gene risk score, we divided patients into high- and low-risk Youden index cutoff values in the training cohort. Survival analysis showed that patients in the high-risk group had a poorer OS than those in the low-risk group. This five-set gene signature was selected by performing survival analysis using the Cox proportional hazard regression model, with the threshold of the *p* value set as 0.05 that accurately predicted five-year OS and separated the high-risk and low-risk patient groups in the in silico training cohort (HR = 4.76, *p* = 0.012; Figure 2A). Additionally, we compared the prognostic performance of the five-gene signature risk score by time-dependent ROC curve analysis and found that the five-gene signature risk score outperformed each individual marker (all *p* < 0.05, Wilcox test; AUC = 0.73, *p* < 0.0001; Figure 2B). Moreover, the five-gene signature can also use a strong prognostic marker for colon (HR = 4.19, *p* < 0.0001, Figure 2C; AUC = 0.72, *p* < 0.0001, Figure 2D) and rectum (HR = 9.87, *p* = 0.003, Figure 2E; AUC = 0.82, *p* < 0.0001, Figure 2F) cancer, individually. Interestingly, the RFS for the patients in the high-risk group was significantly poorer than that for the patients in the low-risk group (hazard ratio (HR) = 3.66, *p* = 0.0006; Figure 2G), and the prediction accuracy value was 69.8% for the high-risk group (Figure 2H).

**Figure 3:**
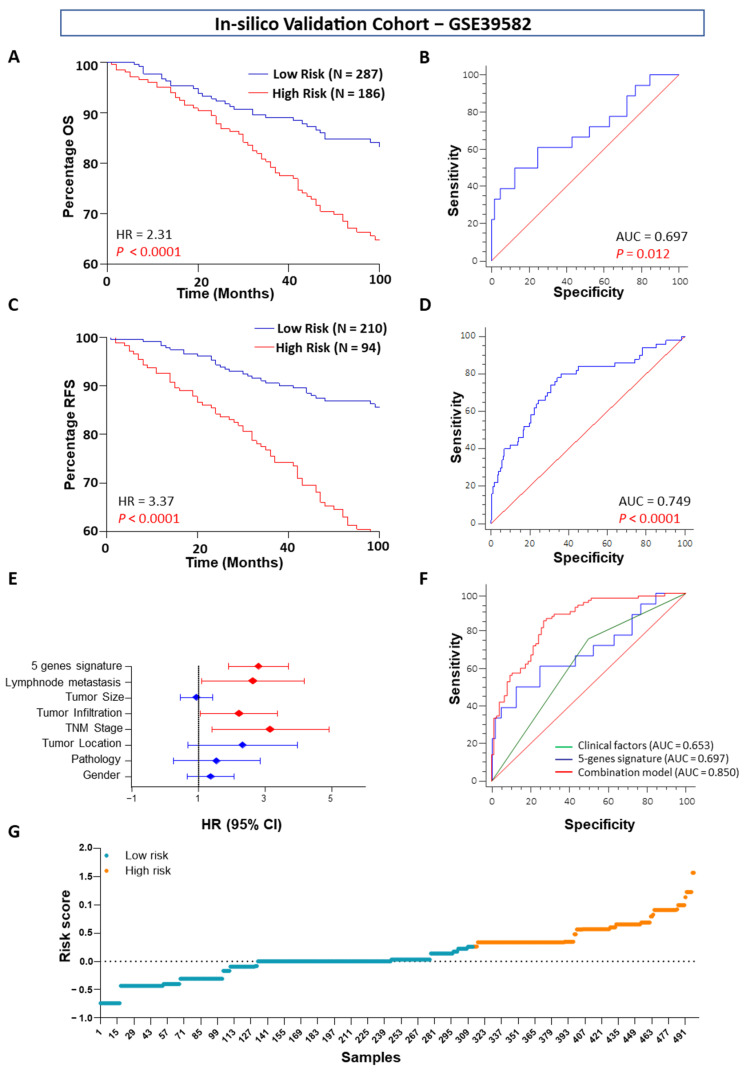
Kaplan–Meier survival plots and ROCs for OS and RFS by five-gene signature scores in the in silico validation cohort (GSE39582). (**A**) Five-gene signature OS for low- and high-risk patients, (**B**) ROC curve of the five-gene signature for OS. (**C**) Five-gene signature RFS curve for low- and high-risk patients. (**D**) ROC curve of the five-gene signature for RFS. (**E**) Estimated HR and 95% CI for the five-gene signature and the clinical factors in univariate Cox potential hazard regression analysis, (**F**) ROC curves achieved with five-gene signature risk scores as well as their combination with the significant clinical factors. (**G**) Estimated risk score between low and high-risk patients group.

The same gene signature was tested in one independent validation cohort to predict OS and RFS. The five-gene signature successfully predicted accurate OS (HR = 2.31, AUC = 0.69) and RFS (HR = 3.37, AUC = 0.74) in the in silico validation cohort GSE39582 (Figure 3A–D). Furthermore, in multivariate Cox regression analysis, the five-gene signature had better predictive ability for OS than all other significant clinical factors (lymph node metastasis and TNM stage) (Figure 3E). ROC analysis revealed that the combination of the five-gene signatures with the clinical risk stratification scheme alone was superior for OS prediction in the training cohort (Figure 3F). The risk scores of the combination model of five selected genes were significantly high for the nonsurviving patients in this validation GEO patient cohort (Figure 3G). Therefore, this five-gene signature may have important application in predicting the OS and RFS of patients with CC.

### 2.2. Signature Validation and Clinical Association in Clinical Cohort

**Figure 4:**
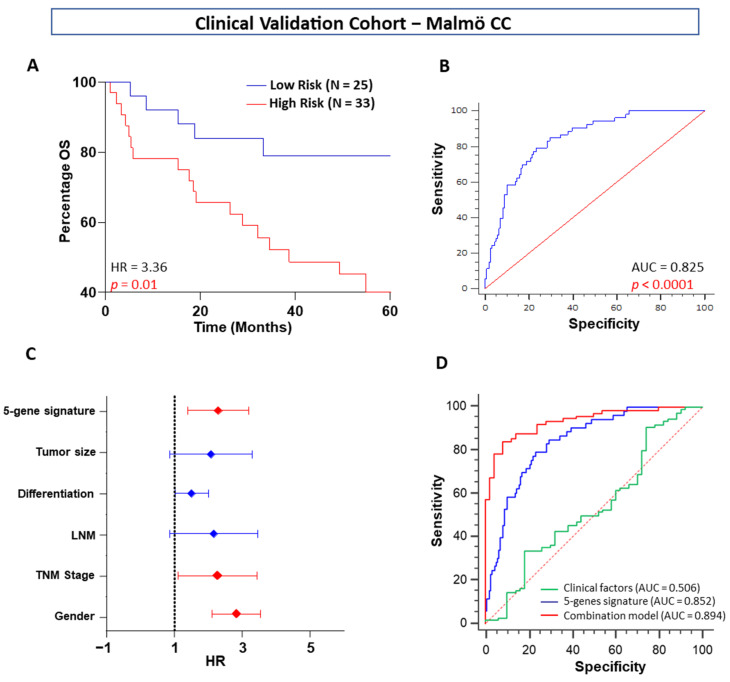
Kaplan–Meier survival plots and ROCs for OS stratified by five-gene signature scores in the patient clinical validation cohort (Malmö CC). (**A**) Five-gene signature OS curve for low- and high-risk patients, (**B**) ROC curve of the five-gene signature for OS. (**C**) Estimated HR and 95% CI for the five-gene signature and the clinical factors in univariate Cox potential hazard regression analysis. (**D**) ROC curves achieved with five-gene signature risk scores as well as their combination with the significant clinical factors.

To validate the prognostic performance of the five-gene risk score, patients in the validation cohort were classified into high- and low-risk groups using the same cutoff obtained from the training cohort in silico training cohort. In the validation cohort, patients with a high-risk score had a worse OS (HR = 3.36, *p* = 0.01, Figure 4A) based on the five-protein immunohistochemistry (IHC) scores. All five protein IHC expression patterns and gene directions were similar in the clinical validation cohort and in the in silico training and validation cohorts. By performing cross-validated time-dependent ROC curves, the area under the respective ROC curves (AUC) was 0.82 (Figure 4B), confirming the prediction accuracy of this model. After adjustment for baseline clinicopathologic factors, the five-gene signature remained a significant predictor of OS in the validation cohort (*p* = 0.01; Figure 4C). We also noted similar results in the in silico validation cohort (Figure 3E). ROC analysis revealed that the combination of the five genes with the clinical risk stratification scheme alone significantly correlated with OS (AUC = 0.89, *p* < 0.001, Figure 4D).

### 2.3. Biomarker Panel Validation in Training and Independent Plasma-Based Clinical Cohorts

**Figure 5:**
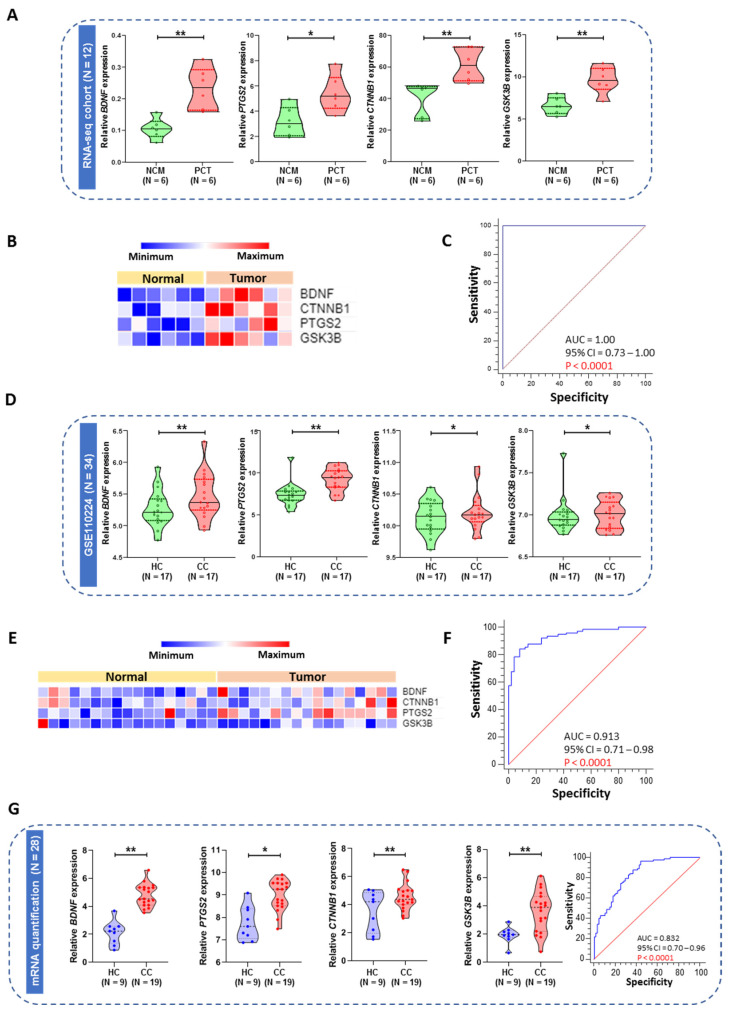
Violin plot of the relative expression of four selected genes (*BDNF*, *PTGS2*, *CTNNB1*, *GSK3B*) in the microarray, RNA-seq, and real-time-based plasma cohorts. (**A**) Relative expression of four genes in the tissue-based RNA-seq clinical cohort. (**B**) Heatmap and unsupervised clustering representation of the differentially expressed four-gene signature in the clinical discovery RNA-seq cohort. (**C**) ROC curve for the discovery cohort for stage I, II, and III samples. (**D**) Relative expression of four genes in the plasma-based microarray in silico training cohort (GSE110224). (**E**) Heatmap and unsupervised clustering representation of the differentially expressed four-gene signature in the plasma-based microarray in silico training cohort. (**F**) ROC curve for the training cohort for stage I, II, and III samples. (**G**) Relative expression of four genes in the plasma-based clinical validation cohort and ROC curve for stage I, II, and III samples. NCM—Normal colon mucosa (matched); PCT—Primary colon tumor (matched); HC—Healthy control; CC—Colon cancer. *p* value * < 0.05, ** < 0.01.

One of the genes (*HPGD*) was eliminated from the five-gene signature due to its very low expression in the tumor samples compared to its matched normal tissue, which makes it not suitable for detection in plasma samples from CRC patients. To develop an mRNA-based signature for the identification of early diagnosis markers, we first interrogated one RNAseq-based mRNA expression profiling result for six colon tumors and its matched normal tissues. We filtered four genes (*BDNF*, *PTGS2*, *CTNNB1*, and *GSK3B*) from the colon tumor and matched normal RNA-seq data, estimated the differences in fold change, and performed a t-test to determine the *p* value representing significant differences. All the genes were significantly upregulated in tumor samples compared with its matched control in the RNA-seq clinical cohort (Figure 5A,B). The detective power of the diagnosis of the robust signature was calculated by ROC curve analysis with an AUC value of 1 (95% CI = 0.73–1.00; *p* < 0.0001) (Figure 5C). The four-gene signature identified from the mRNAseq data was first measured in the plasma-based microarray in silico training cohort (GSE110224; 17 CRC cases and 17 healthy controls). The selected four genes were significantly upregulated in CRC patients compared with healthy control plasma samples in this training cohort (Figure 5D,E). We calculated the coefficients and prepared a risk score from this cohort. Multivariate logistic regression was subsequently used to build a scoring formula for quantifying the early detection of CC, and the AUC of the four-gene panel was 0.91 (95% CI = 0.71–0.98; *p* < 0.0001; Figure 5F). Next, we assessed the robustness of this four-gene panel in an independent plasma-based clinical validation cohort (19 CRC cases and 9 healthy controls). We observed a similar significant upregulation for all four genes in CRC patients compared to healthy control plasma samples in our clinical validation cohort (Figure 5G). Subsequently, we applied the same coefficients in the clinical validation cohort, which yielded a robust AUC value of 0.83 (95% CI = 0.70–0.96; *p* < 0.0001) in stage I–III patients (Figure 5G). The individual AUCs were superior in the clinical validation cohort than in the in silico training and validation cohorts (Figure 2A and Figure 3A).

## 3. Discussion

In our quest to develop a robust CRC prognostic signature, we successfully developed a five-gene signature that achieved excellent predictive values in OS and tumor recurrence in patients with stage I, II, and III CRC, which were validated in one tissue-based independent clinical cohort. Furthermore, when compared with the other clinicopathologic risk factors, our five-gene classifier remained the strongest prognostic indicator. To further highlight the clinical significance of our findings, our five genes significantly stratified patients from all clinical cohorts into high- and low-risk subgroups robustly.

The current TNM staging system based on The American Joint Committee on Cancer: the 7th edition (AJCC 7th edition) is closely associated with patient prognosis [5]. The univariable and multivariable Cox regression analyses in our study consistently showed that tumor stage was a significant prognostic factor in both the training and validation cohorts. Therefore, stratification analysis was performed to investigate whether these five-gene signatures were independent of tumor stage. The results showed that it could also discriminate the high-risk patients from the stratified groups in the in silico discovery, training, validation, and clinical validation cohorts. One important question should be mentioned here: the ethnicity of the in silico and clinical cohorts differed in this study. Successful validation indicated that our gene signature was not only across populations but was also independent of tumor stage, as our signature was independent of tumor stage. Thus, the conclusions of our analyses were convincing.

Recent studies have shown that right- and left-sided CRCs have different epidemiologic and histological characteristics as well as underlying biological mechanisms [14,15]. However, when we stratified the patients by tumor location in our clinical cohort, we found that our five-gene signature could not discriminate high-risk patients from the subgroup of rectal carcinoma. This result indicated that our signature may apply to left-sided or right-sided CCs. Notably, there were very few patients with rectal carcinoma, so bias might have affected the stratification analysis. It is necessary to enlarge the sample size to generate more reliable results. Previously, a 13-gene-based classifier was reported to predict tumor recurrence in patients with stage II CRC [16]; however, in this study, our five-gene classifier performed significantly better and illustrated its ability to predict OS and recurrence not only in patients with stage I and II CRC but also in patients with stage III CRC.

Although we did not perform a direct comparison, the OS and recurrence prediction values for our five-gene signature were superior to gene expression-based signatures offered by the ColoPrint and Oncotype DX assays [17]. It would also be interesting to validate our markers or stratify them based on CDX2 as well as recently published immune scores in the future. An ideal prognostic classifier for CRC risk prediction should be robust, reproducible, and most importantly, potentially feasible in FFPE materials, which would eliminate the need to plan and invest methodologies to collect and preserve fresh-frozen tumor specimens. Our five-gene classifier successfully overcomes these barriers, as evidenced by its superior performance and independent validation in a cohort of FFPE specimens. The availability of ideal prognostic and predictive biomarkers is essential for achieving clinical goals in refining therapeutic decisions and thereby improving the survival and quality of life of patients with CRC.

ROC analysis showed that our five-gene signature was superior to tumor stage for prognostic evaluation. To further improve the prognostic prediction ability, we combined the five-gene risk model with TNM staging and lymph node metastasis. There was very little difference between the combined model and our gene signature, indicating that our five-gene signature could yield results alone or in combination with TNM staging and lymph node metastasis information. As a result of poor reproducibility, most established signatures have not been used clinically for prognostic prediction in CRC. The reasons for poor reproducibility are manifold. In early studies, small sample series and lack of validation in independent samples limited the strength of the conclusions. In addition, some gene signatures use too many genes for the construction of a model, which inhibits their clinical utility. Importantly, most studies of gene signatures are retrospective; good reproducibility is still hampered by the lack of validation in prospective multicenter studies [18,19].

As a new diagnostic test for determining the likelihood of recurrence in stage II CRC patients after surgical resection, the OncotypeDX colon cancer assay has been commercially available worldwide since 2010 [17,20]. The results indicated that this five-gene signature might be a useful tool for the management of CRC patients. As our study was retrospective, its reliability still needs further validation in a large prospective study.

The innovation of our research rests on the following aspects. First, the AUC of our five-gene signature was fairly large (>0.75), indicating good prognostic ability. Second, our study is a relatively systematic examination of prognostic gene signatures in CRC. Third, our study has several strengths related to the study design and analytical methods. The five-gene classifier was validated in independent in silico datasets as well as one independent population and IHC-based clinical cohort. As we developed a “risk prediction model” using our five-gene signature, the scores can be readily applied to independent, future prospective cohorts. Our assay also demonstrated effectiveness in FFPE tissues. Our signature is derived from a common cancer-related gene that plays a major role in cancer cell development, apoptosis, and metastasis; thus, this signature is closely related to cancer cell growth, apoptosis, and metastasis and should be suitable for prognostic assessment.

Although we did not have access to blood specimens in this study for prognostic assessment, we feel encouraged that given the stability and relative abundance of five- genes in circulation, it is very likely that our five-gene signature may eventually be translated into a blood-based, predictive, surveillance assay as OS and RFS. Furthermore, in this study, we proposed an mRNA biomarker panel for the detection of CRC in human plasma by measuring four mRNAs retrieved from bioinformatics analysis and comparing their sensitivities and specificities in one RNAseq and plasma-based GEO microarray cohort. We first investigated the upregulated four genes (*BDNF*, *PTGS2*, *CTNNB1*, and *GSK3B*) in the tumor sample expression profile in CC patient tissue using RNAseq data. In our recent study, we showed that *BDNF* with *CYSLTR1* and *CD66B* together or individually identified the high-risk group or were used as strong prognostic markers in CRC patients, and these markers had a significant role on CC progression in the mice xenograft model [13,21]. Whereas, another earlier study from our group showed that non-canonical WNT5A signaling in CC cells have an opposite effect than canonical β-catenin signaling on 15-PGDH (*HPGD*) expression due to its ability to inhibit β-catenin (*CTNNB1*) in CC cells [22]. As shown in another study, even patients who were defined as having a poor prognosis had high nuclear β-catenin and COX-2 expression and low 15-PGDH expression [23]. In our earlier studies, we provided first evidence that LTC_4_, via CysLT_2_R signaling, can induce 15-PGDH expression through the activation of the JNK/AP-1 pathway, and this activation in turn prompts the differentiation of CC cells [24]. In a follow-up study, we revealed that the induction of 15-PGDH by LTC_4_/CysLT_2_R signaling axis in CC cells downregulated GLI1 and induced differentiation in CC cells [25].

From a clinical standpoint, early detection is a vitally important factor for therapeutic decision making, especially in CRC, due to the lower mortality rate and poor symptoms in the early stage. If patients can be diagnosed in the early stage of cancer, before lymph node metastasis, then the surgical resection and design of the treatment strategy could be improved. An ideal biomarker should be noninvasive, accurate, inexpensive, specific, sensitive, reliable, and reproducible [26,27]. Consistent with this idea, body fluids are commonly used materials in the diagnosis of multiple human diseases [26,28]. In particular, plasma has significant advantages in reflecting gastrointestinal diseases [29,30]. To date, microRNAs (miRNAs) have been widely reported to be biomarkers in body fluids [31]. Our data indicated that the expression patterns of all differentially upregulated four-gene signatures in patient plasma samples of clinical cohorts were consistent with the results of discovery and training analysis. The relative expression levels of four selected mRNAs in CRC plasma samples were higher than those in healthy control plasma samples for the plasma-based clinical cohort. CRC plasma mRNA levels were also associated with major clinicopathological factors. All these results strongly suggested that the selected mRNAs may be novel potential CC-associated biomarkers. Due to the low sensitivity or specificity of the existing blood biomarkers of CRC, a panel approach of combinations will allow us to pursue a more precise diagnosis. As shown in Figure 5G, we evaluated the combination of our four gene indices using a logistic model. In the plasma from the CRC patients cohort, the AUC of the combined use of our four genes was up to 0.83, with sensitivity = 59.63% and specificity = 96% (Figure 5G). These results mean that the diagnosis of the selected novel genes signature will have satisfactory specificity and sensitivity. Nevertheless, to confirm the specificity of this method, a larger cohort of patients should be analyzed in the future.

## 4. Materials and Methods

### 4.1. Patient Cohorts

This study included multiple clinical cohorts with a total of 1850 patients. These cohorts include patients from the publicly available dataset from GSE44076 (N = 246), GSE44861 (N = 92), GSE41258 (N = 240), GSE37364 (N = 65), GSE110224 (N = 34), The Cancer Genome Atlas (TCGA-COADREAD; N = 404), and GSE39582 (N = 585) as well as clinical validation tissue-based cohorts of Malmö-CC (N = 144), Malmö-CC RNA-seq (N = 12), and plasma from Linköping-CRC cohort (N = 28) (Table 1). The first clinical cohort (cohort 1 as the prognostic validation cohort) comprised formalin-fixed, paraffin-embedded tissues (matched normal and tumor samples) from 72 patients who were enrolled at Malmö Hospital during 1990 and were followed up after 120 months [32]. The second clinical cohort (cohort 2 as the diagnostic cohort) included plasma from 19 CRC patients and 9 healthy control plasma samples [33]. The studies were conducted in accordance with the Declaration of Helsinki, and the respective institutional review boards approved the study.

### 4.2. Identification of the mRNA Signature from Microarray and Genome-Wide RNA Sequencing Data

Four public datasets were analyzed in the discovery phase *for marker selection*: CRC microarray data from GSE44076 (N = 246), GSE44861 (N = 92), GSE41258 (N = 240), and GSE37364 (N = 65) from the Gene Expression Omnibus (GEO). One RNA sequencing dataset from TCGA (N = 404) and microarray dataset GSE39582 (N = 585) from GEO were used as the in silico training and validation cohort, respectively. The discovery phase datasets were chosen according to the availability of the sample sizes and types of tissues. The GSE444076 microarray dataset contains mRNA expression of fresh frozen (FF) tissue from healthy control mucosa (HCM, N = 50), matched normal colon mucosa (NCM, N = 98), and matched primary colon tumor (PCT, N = 98), which contained a satisfactory number of sample size in stage II and III CRC in each group (Table 1). Whereas, the GSE41258 dataset contains a significant amount of formalin-fixed paraffin-embedded tissue with stage I, II, III, and IV samples. We also used two other datasets (GSE44861 and GSE37364), which contain a sufficient number of matched NCM and matched PCT, but other clinical information was missing in both the datasets. The TCGA-COADREAD (RNA-seq) and GSE39582 (microarray) datasets were used as in silico training and validation cohorts because both the datasets contain a significant number of stage I, II, and III samples with all the clinical information (including OS and RFS information, Table 1). A total of five cancer-related genes were selected based on the Gene Ontology and cancer hallmark database (Figure S3). The in silico discovery cohort was used to identify differentially regulated cancer-related gene signatures between adjacent normal and tumor samples in CRC patients. The mRNA expression levels, measured by reads per million mRNA mapped (RPM), were first log2 transformed. We checked the expression of five previously selected genes that reached significance (*p* = 0.05) and log2-fold change > ±1. The differentially regulated genes were represented as upregulated and downregulated in the volcano plot for all the datasets (Figure S4). We used these five genes to build the model using the backward elimination Wald test to check the feasibility of combining the five genes in the prognostic model. Differential mRNA expression analysis was subsequently performed between normal and tumor tissues at five-year survival using the Wilcoxon signed-rank test. For the in silico validation of identified mRNAs, we analyzed one additional independent cohort, GSE39582 (death = 112, alive = 361), and the TCGA cohort (death = 75, alive = 249). The mRNA expression profiles were normalized using the robust multiarray average (RMA) algorithm in R. We downloaded preprocessed data from GEO using the Bioconductor package “GEOquery.” Using multivariate Cox regression analysis, we calculated risk scores and assessed the prognostic performance of the mRNA signature-based survival analysis using Youden’s J Statistic index association value of the predicted risk scores in each dataset as the cutoff.

### 4.3. Immunohistochemistry

The paraffin-embedded samples were cut into 4-µm sections, deparaffinized, and incubated in 10 mM citrate buffer (pH 6.0, S1699; Dako, Glostrup, Denmark) for 20 min in a microwave oven. Blocking buffer (Dako) with 10% fetal bovine serum in PBS was added to the slides for 10–20 min, and then, a specific serum-free protein block (Dako) was added for 30 min at RT. After washing, the sections were stained using Dako Autostainer Plus18 with the following primary antibodies: rabbit monoclonal antibody against BDNF (dilution 1:1000, pH 6. RT, 1 h; ab108319; Abcam, Cambridge, UK), rabbit polyclonal antibody against 15-PGDH (dilution 1:500, pH 6. RT, 1 h; Novus Biologicals, Cambridge, UK), mouse monoclonal antibody against β-catenin (dilution 1:500, pH 6. RT, 1 h; BD Transduction Laboratories, Becton, NJ, USA), rabbit polyclonal antibody against GSK-3β (dilution 1:50, pH 6. RT, 1 h; Cell Signaling Technology, Danvers, MA, USA), and rabbit polyclonal anti-COX-2 (dilution 1:150, pH 6. RT, 1 h; ab52237; Abcam, Cambridge, UK). The secondary antibody Envision+ System-HRP-labeled polymeric anti-rat/anti-rabbit antibody (Dako) was visualized using 3,3′-diaminobenzidine (DAB) substrate (Vector Laboratories Inc., Burlingame, CA, USA) and counterstained with hematoxylin.

Four genes (*BDNF*, *PTGS2*, *GSK3B*, and *CTNNB1*) were significantly upregulated, and one gene (*HPGD*) was significantly downregulated in primary tumor tissues compared with adjacent normal tissues in all five in silico datasets. A correlation matrix and heatmap were generated for the selected markers, and we found a significant correlation between the mRNA expression of *BDNF* and 4 other genes, *CTNNB1*, *PTGS2*, *GSK3B* (β-catenin, COX-2, and GSK-3β respectively), and *HPGD* (15-PGDH) in primary tumor tissues compared to adjusted colon mucosa and colon mucosa from healthy donors.

We compared gene expression between cancer and normal tissue from a training TCGA dataset (N = 324) and built a prognostic model and calculated the risk score of selected genes in stage I, II, and III CRC patient populations. We performed OS and RFS prognostic analyses based on the in silico data and used an mRNA-based prognostic model to predict the risk score for CRC patients using univariate and multivariate analyses after including the significant clinical factors in the in silico training and validation cohorts. By combining all these results, we extracted the five-gene signature and suggested it as an independent predictor panel with the best prognostic assessment.

For validation, the selected prognostic gene signatures in our clinical cohort from Malmö Hospital (a TMA with 58 CC patients from both normal and tumor areas, stage I–III) were analyzed using IHC. Human tumor tissues and their normal matched pair colon tissues were stained against BDNF, β-catenin, COX-2, GSK-3β, and 15-PGDH, and the results from the evaluation of the protein levels were consistent with in silico data.

### 4.4. RNA Isolation and mRNA Expression Analysis from Tissue and Plasma Samples

Six colon adenoma patients and six matched normal tissues were collected from the Malmö Cancer Hospital. Those adjacent normal tissues were confirmed after performing histopathology by a trained pathologist. Plasma samples from 19 CC patients and 9 normal healthy controls were collected for validation as diagnostic markers. Anti-coagulated blood was collected in EDTA tubes and centrifuged at 1600× *g* for 10 min at 4 °C. The clear upper layer plasma was collected and recentrifuged at 16,000× *g* for 10 min at 4 °C to remove residual cell pellets. After that, cell-free plasma was collected and preserved in 1 mL Qiazol Reagent (Qiagen, Germantown, MD, USA) before storage at −80 °C.

Total RNA was extracted from the colon tumor and adjacent normal samples, which was followed by quantification using a bioanalyzer (Agilent Technology, Santa Clara, CA, USA). The extracted RNA was sent to the Lund University sequencing facility for transcriptome analysis. A sequencing library was prepared using a TruSeq Targeted RNA Expression Kit (Illumina, San Diego, CA, USA), and data were filtered using the Trimmomatic package. The mRNA expression levels, measured by reads per million mRNA mapped (RPM), were first log2 transformed.

Total RNA from the plasma samples was isolated using an RNeasy mini kit (QIAGEN, Germantown, MD, USA). mRNA expression analysis was performed using an Agilent real-time PCR system (Agilent Technology, Waltham, MA, USA). Specific TaqMan probes, rather than the more commonly used gene-specific primers, of selected genes were purchased from Thermo Scientific. qRT–PCR assays were conducted using an RNA reverse transcription kit (Applied Biosystems, Waltham, MA, USA) using a Thermosphere probe mix kit. The relative expression of mRNA was determined by the 2-Δct method using β-actin as a normalizer, as described previously. All results are expressed as the mean ± SD of three independent experiments.

To evaluate whether our suggested panel can be considered an accurate indicator of early diagnosis in CRC patients, we conducted a study in which we analyzed the mRNA level of the selected markers in plasma from a plasma-based CRC patient cohort (N = 28). We built a logistic model and calculated the risk score for the four-gene signature (only the upregulated genes were selected due to the lower concentration of mRNA in plasma samples) for use as an early diagnosis marker panel using tissues and plasma samples from CRC patients, after comparing the gene expression between healthy mucosa and primary CC tumors and healthy controls plasma with cancer patient plasma samples. The risk score will be validated in a plasma-based clinical cohort using RT-PCR methods for the four-panel gene signature.

### 4.5. Statistical Analysis

Statistical analyses were performed using IBM SPSS version 20 (Chicago, IL, USA), MedCalc version 18 (Ostend, Belgium), GraphPad Prism version 8.0 (La Jolla, CA, USA), and R 3.2.4. Statistical differences between mRNAs and various clinicopathologic factors were determined by the χ2 test. The Benjamini–Hochberg method was used to correct for multiple hypothesis testing wherever applicable. All statistical tests were two-sided, and a *p*-value of <0.05 was considered significant. OS was defined from the day of surgery to death or the end of follow-up and was analyzed by the log-rank test. We performed receiver operating characteristic (ROC) curve analysis to evaluate the predictive power of the selected gene signature. All five mRNA expression values derived from the transcriptome datasets were used to build an overall survival classifier (OSC) using Cox proportional hazard regression. The risk scores derived from the five-gene OSC Cox model were used to plot the area under the curves (AUC). The risk scores were calculated using the formula derived from the Cox model. The risk score was validated using the immune reactivity score from IHC slides in a tissue-based cohort for OS prognostic assessment. To evaluate the association of protein expression in CC tissue with OS, univariate and multivariate Cox proportional hazards regression models were applied, and hazard ratios (HRs) together with 95% confidence intervals (CIs) were calculated to determine the risk of death or cancer recurrence. The multivariate model was adjusted for established prognostic factors such as age, sex, lymph node metastasis (LNM), tumor-node-metastasis (TNM) stage, and tumor size. All patients with incomplete or missing cores were excluded from the analysis. To plot the Kaplan–Meier curves, we dichotomized the patients into low- or high-risk groups based on Youden index-derived cutoff values (X-tile software 3.6.1, Yale School of Medicine). Additionally, we performed univariate and multivariate Cox proportional hazard regression models using clinicopathologic variables and OSC to calculate estimated hazard ratios (HRs). Only the significant variables in the univariate model were used to perform the multivariate analysis. The differences in mRNA levels between plasma of CRC patients and healthy control plasma samples were assessed using the t-test for paired data. The correlations between mRNA levels and clinicopathological factors were further analyzed by one-way analysis of variance (ANOVA). We performed receiver operating characteristic (ROC) curve analysis to evaluate its diagnostic value. All four mRNA expression values derived from RT-PCR were used to build an mRNA signature early diagnosis classifier using a logistic regression model. The risk scores derived from the four-mRNA signature logistic model were used to plot the area under the curves (AUCs). Additionally, we performed univariate and multivariate logistic regression models using clinicopathologic variables and mRNA signatures. Only the significant variables in the univariate model were used to perform the multivariate analysis. Experimental reproducibility was determined by the Pearson correlation test.

In this study, the limitations concerning clinical sample size can be ignored because the Swedish ethnic group has a very small population size, and that the study was well backed up with a large number of CRC samples from different public databases. Another limitation is that the study contains only one ethnic population for independent validation for the prognostic and diagnostic markers. However, the fact that our study is one of the first to perform that the same genes could be used as prognostic as well as diagnostic markers and significantly separate the high-risk group from the low-risk group and provide insights for early diagnosis of CRC patients might make it very useful.

## 5. Conclusions

In conclusion, we provide novel evidence that this five-gene signature can effectively stratify patients with stage I, II, and III CRC into high- and low-risk groups based upon clinical outcomes, thereby offering significantly improved prognostic biomarker potential compared with the currently used clinicopathologic risk factors. Moreover, it outperforms other known gene signatures, indicating that the five-gene signature may be a useful tool for clinicians and will facilitate the personalized management of CRC patients. Furthermore, the five-gene signature is an independent predictor of tumor recurrence, which, in combination with TNM stage and lymph node metastasis, offers an easy-to-translate and facile assay for personalized risk assessment in stage II/III CRC patients. Additionally, we found that all four selected mRNAs were increased in CRC tissues and patient plasma, which makes it easy to use in clinical practice. As a result, *BDNF* may be a valuable blood-based biomarker, either alone or in combination, to screen for CRC. Further validation of this signature in multicenter CRC cohorts might lead to the development of an affordable, noninvasive diagnostic and population screening assay for CRC patients.

## Figures and Tables

**Table 1:** Distribution of clinical and pathological covariates of in silico and clinical datasets.

Datasets	Sample Type	Tissue Types	Data Types	Platform	Age (Mean ± SD)	Gender			Anatomical Location				TNM Stage			
						Male (N)	Female (N)	Missing (N)	Left (N)	Right (N)	Right (N)	Missing (N)	Stage I	Stage II	Stage III	Stage IV
*GSE44076*	HCM (N = 50)	FF	Tissue mRNA	Microarray	62.50 ± 14.20	27	23	-	23	27	27	-	-	-	-	-
	NCM (N = 98)				70.54 ± 9.02	71	27	-	60	38	38	-	-	-	-	-
	PCT (N = 98)				70.54 ± 9.02	71	27	-	60	38	38	-	-	42	56	-
*GSE44861*	NCM (N = 46)	FF	Tissue mRNA	Microarray	Missing	Missing			Missing				Missing			
	PCT (N = 46)															
*GSE41258*	NCM (N = 54)	FFPE	Tissue mRNA	Microarray	61.70 ± 16.34	24	21	9	25	20	20	9	-	-	-	-
	PCT (N = 186)				63.54 ± 13.94	98	87	1	108	77	77	1	28	50	49	58
*GSE37364*	NCM (N = 38)	FF	Tissue mRNA	Microarray	Missing	Missing			Missing				Missing			
	PCT (N = 27)															
*TCGA COADREAD*	NCM (N = 51)	FF	Tissue mRNA	RNA-seq	69.2 ± 14.16	25	26	-	25	21	21	5	-	-	-	-
	PCT (N = 353)				64.36 ± 13.16	193	160	-	179	162	162	12	56	135	111	51
*GSE39582*	NCM (N = 19)	FF	Tissue mRNA	Microarray	68.16 ± 10.12	12	7	-	14	5	5		-	-	-	-
	PCT (N = 566)				66.91 ± 13.27	310	256	-	337	227	227	2	35	268	207	56
*Malmö-CC*	NCM (N = 72)	FFPE	Tissue protein	IHC	69.52 ± 12.61	33	39	-	34	26	26	15	-	-	-	-
	PCT (N = 72)				69.52 ± 12.61	33	39	-	34	26	26	15	9	28	21	14
*Malmö-CC RNA-seq*	NCM (N = 6)	FFPE	Tissue mRNA	RNA-seq	64.17 ± 8.46	3	3	-	Missing				-	-	-	-
	PCT (N = 6)				64.17 ± 8.46	3	3	-					1	2	2	1
*GSE110224*	HC (N = 17)	Plasma	Circulating mRNA	Microarray	Missing	Missing			Missing				Missing			
	CC (N = 17)															
*Linköping-CRC cohort*	HC (N = 9)	Plasma	Circulating mRNA	Real-time qPCR	69.37 ± 9.28	6	3	-	Missing				-	-	-	-
	CC (N = 19)				70.93 ± 9.39	13	6	-					6	8	5	-

HCM—Healthy control mucosa; NCM—Normal colon mucosa (matched); PCT—Primary colon tumor (matched); HC—Healthy control; CC—Colon cancer; FF—Fresh frozen; FFPE—Formalin fixed paraffin embedded; IHC—Immunohistochemistry.

## Data Availability

The datasets used and/or analyzed during the current study are available from the corresponding author on request.

## Supplementary Materials

The following are available online at https://www.mdpi.com/article/10.3390/ijms23020793/s1.
