# Supplementary material for: Identification of a Novel Five-Gene Signature as a Prognostic and Diagnostic Biomarker in Colorectal Cancers
Source: Int J Mol Sci. 2022 Jan 12;23(2):793. doi: 10.3390/ijms23020793 (PMC8776147; doi:10.3390/ijms23020793)
Supplement: Supplementary file 1 [file ijms-23-00793-s001.zip › ijms-1526697-supplementary.pdf]

Supplementary material

Identification of a Novel Five-Gene Signature as a Prognostic and Diagnostic Biomarker in Colorectal Cancers

Souvik Ghatak, Syrina F. Mehrabi, Lubna M. Mehdawi, Shakti Ranjan Satapathy and Anita Sjölander *

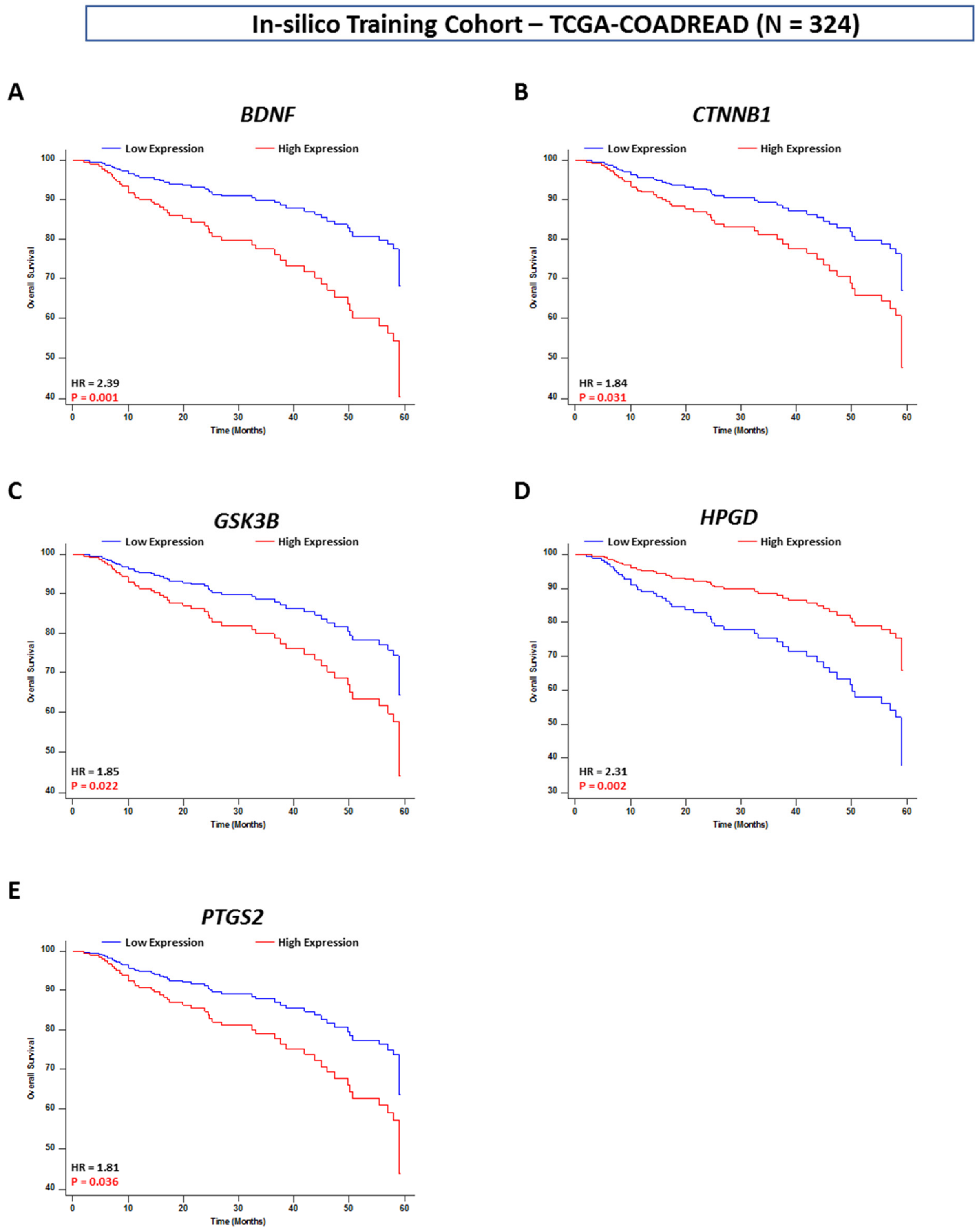

Figure S1. Kaplan–Meier survival plots for overall survival stratified by cox-ph univariate analysis for *BDNF* (A), *CTNNB1* (B), *GSK3B* (C), *HPGD* (D) and *PTGS2* (E) in silico training cohort (TCGA-COADREAD).

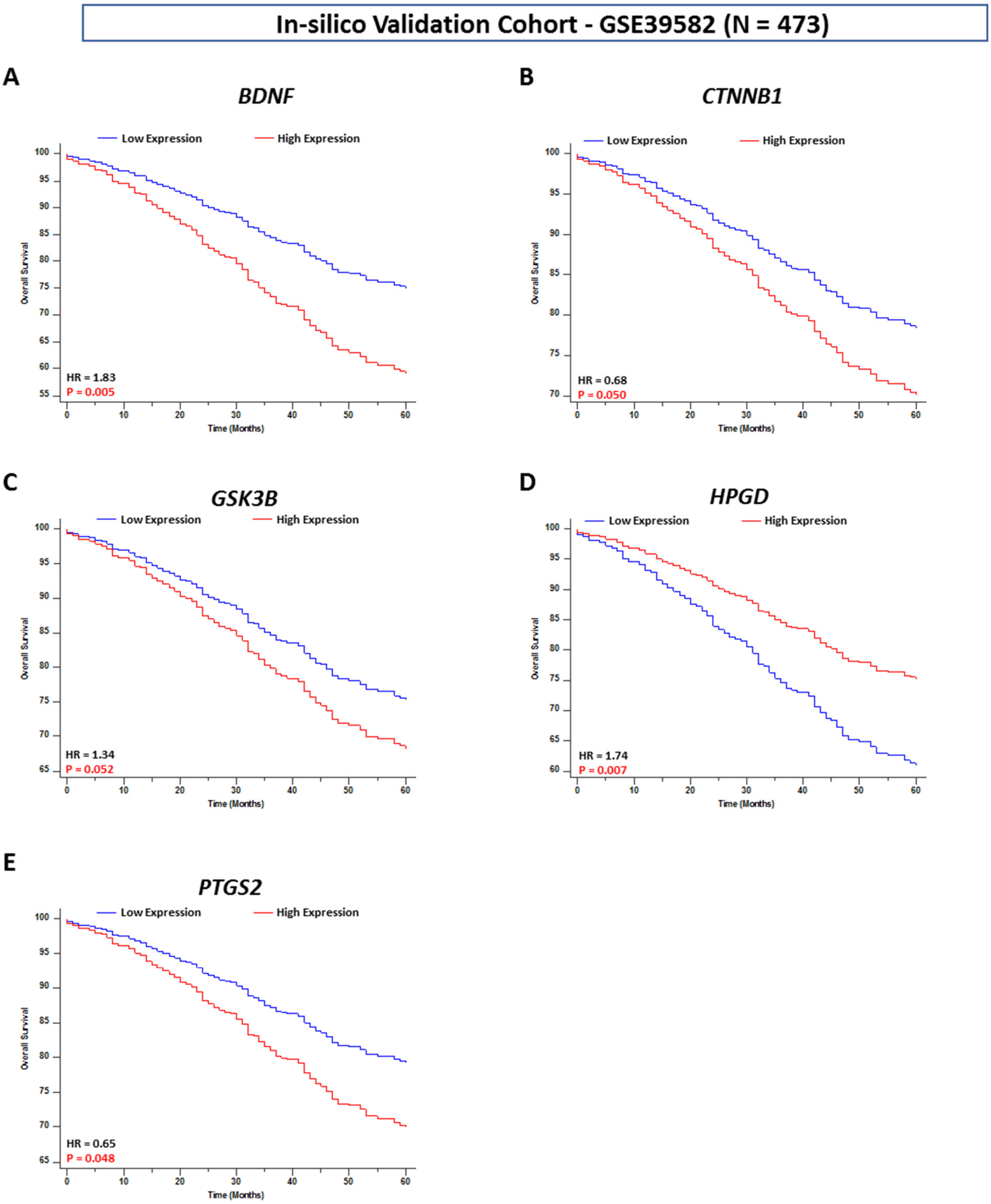

Figure S2. Kaplan–Meier survival plots for overall survival stratified by cox-ph univariate analysis for *BDNF* (A), *CTNNB1* (B), *GSK3B* (C), *HPGD* (D) and *PTGS2* (E) in silico validation cohort (GSE39582).

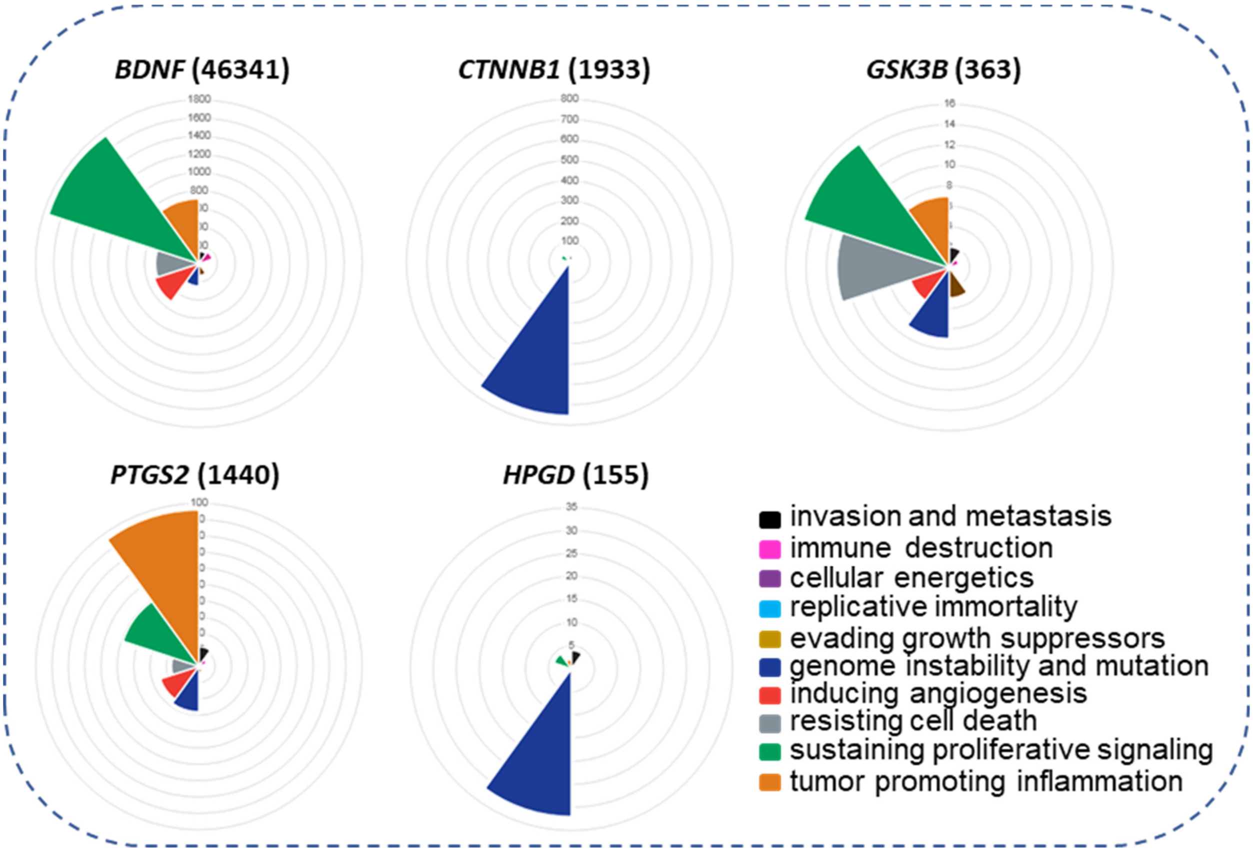

Figure S3. The role of *BDNF*, *CTNNB1*, *GSK3B*, *PTGS2* and *HPGD* in different cancer hallmarks based on the different datasets and publications (based on Cancer Hallmarks Analytics Tool (CHAT); http://chat.lionproject.net).

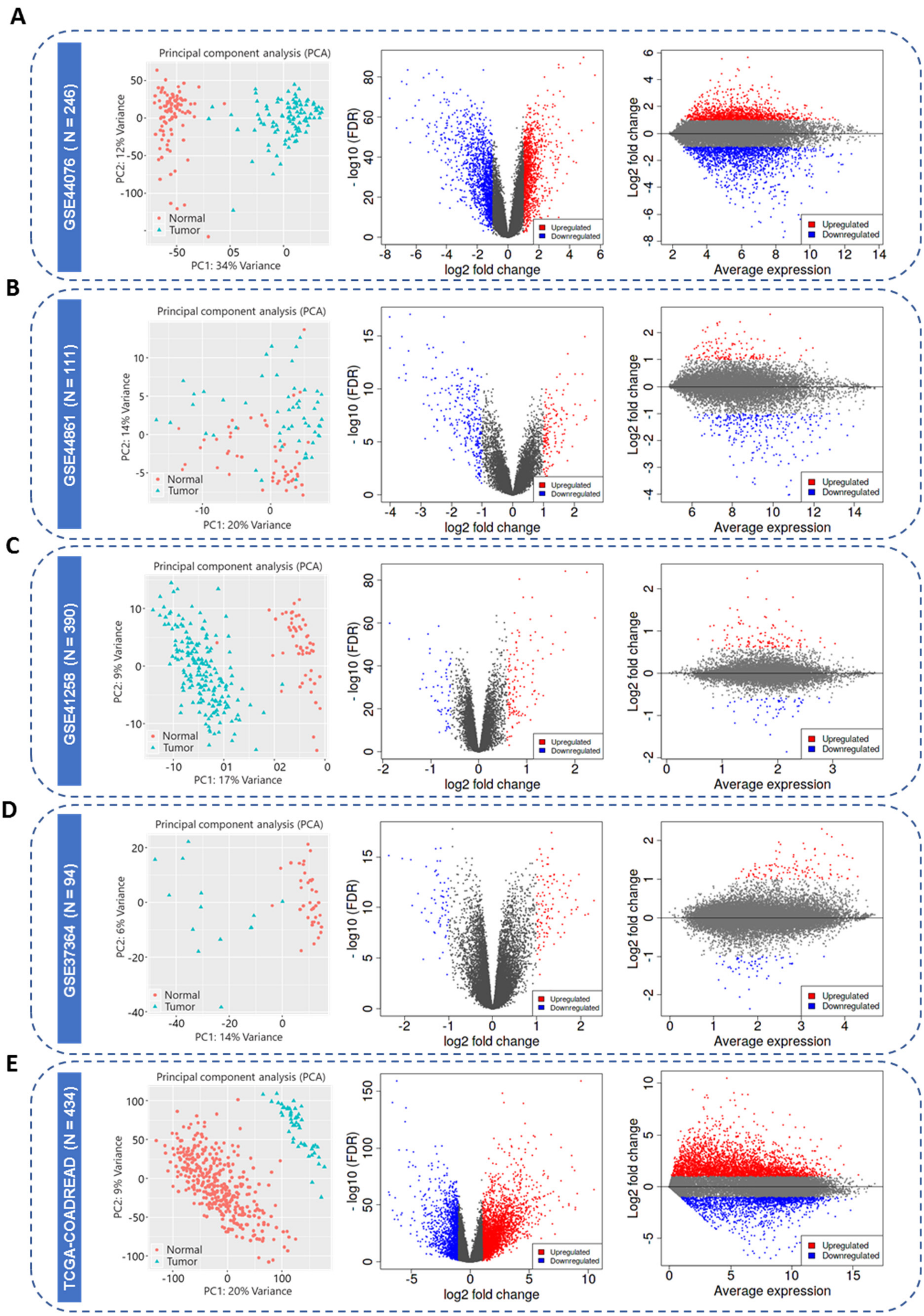

Figure S4. PCA and volcano plot of in silico discovery and training microarray and RNA-seq datasets. (A) GSE44076, (B) GSE44861, (C) GSE41258, (D) GSE37364, (E) TCGA-COADREAD.
